# Early Regional Patterning in the Human Prefrontal Cortex Revealed by Laminar Dynamics of Deep Projection Neuron Markers

**DOI:** 10.3390/cells12020231

**Published:** 2023-01-05

**Authors:** Janja Kopić, Alisa Junaković, Iva Salamon, Mladen-Roko Rasin, Ivica Kostović, Željka Krsnik

**Affiliations:** 1 Croatian Institute for Brain Research, School of Medicine, University of Zagreb, Salata 12, 10000 Zagreb, Croatia; 2 Department of Neuroscience and Cell Biology, Robert Wood Johnson Medical School, Rutgers University, 675 Hoes Lane West, Piscataway, NJ 08854, USA; 3 School of Graduate Studies, Rutgers University, New Brunswick, NJ 08854, USA

**Keywords:** human fetal frontal lobe, postmigratory projection neurons, transcription factors, regional cell aggregation, subplate, transient compartments

## Abstract

Early regional patterning and laminar position of cortical projection neurons is determined by activation and deactivation of transcriptional factors (TFs) and RNA binding proteins (RBPs) that regulate spatiotemporal framework of neurogenetic processes (proliferation, migration, aggregation, postmigratory differentiation, molecular identity acquisition, axonal growth, dendritic development, and synaptogenesis) within transient cellular compartments. Deep-layer projection neurons (DPN), subplate (SPN), and Cajal–Retzius neurons (CRN) are early-born cells involved in the establishment of basic laminar and regional cortical architecture; nonetheless, laminar dynamics of their molecular transcriptional markers remain underexplored. Here we aimed to analyze laminar dynamics of DPN markers, i.e., transcription factors TBR1, CTIP2, TLE4, SOX5, and RBP CELF1 on histological serial sections of the human frontal cortex between 7.5–15 postconceptional weeks (PCW) in reference to transient proliferative, migratory, and postmigratory compartments. The subtle signs of regional patterning were seen during the late preplate phase in the pattern of sublaminar organization of TBR1+/Reelin+ CRN and TBR1+ pioneering SPN. During the cortical plate (CP)-formation phase, TBR1+ neurons became radially aligned, forming continuity from a well-developed subventricular zone to CP showing clear lateral to medial regional gradients. The most prominent regional patterning was seen during the subplate formation phase (around 13 PCW) when a unique feature of the orbitobasal frontal cortex displays a “double plate” pattern. In other portions of the frontal cortex (lateral, dorsal, medial) deep portion of CP becomes loose and composed of TBR1+, CTIP2+, TLE4+, and CELF1+ neurons of layer six and later-born SPN, which later become constituents of the expanded SP (around 15 PCW). Overall, TFs and RBPs mark characteristic regional laminar dynamics of DPN, SPN, and CRN subpopulations during remarkably early fetal phases of the highly ordered association cortex development.

## 1. Introduction

The neocortex is a laminated structure consisting of six neocortical layers that develop in a birth-date-dependent “inside-out” transient lamination pattern. The transient lamination serves as a structural framework for the establishment of first regional differences in the developing neocortex that is especially prominent in primates in the context of areal neuronal organization [1,2,3,4,5,6,7,8,9,10]. This unique organization of transient neocortical laminas and molecular identities of early-born neurons in humans [11,12,13,14,15,16,17] commences during the transitional period between embryonic and fetal life, around 8 PCW. The cellular events in each cortical region are proliferation, migration [18], postmigratory differentiation [19,20,21], acquisition of molecular identity, axonal and dendrite development, synaptogenesis, and ultimately, plasticity [5,10,22]. The molecular specification of neurons and their laminar fate largely depends on the transcriptional and post-transcriptional mechanisms that are pre-programmed during the neuronal prenatal birth date. Specifically, transcription factors (TFs) and RNA binding proteins (RBPs) are two protein subgroups that play major roles in transcriptional and post-transcriptional control of the developing neocortex in mammals, respectively [23,24,25].

A key cellular event within each cortical region during the course of human corticogenesis is the formation of neocortical compartments that will ultimately lay a foundation for the later mature neocortical circuits [1,7,8,9,26,27]. However, the laminar organization of the prenatal human brain does not fully match the laminar organization of the postnatal brain. This anatomical disparity is due to the embryonic and fetal neocortical laminas that serve the role of transient compartments during neocortical development. Specific molecular and cellular events that underlie prenatal cortical lamination and regional organization can be revealed by histological staining [1,7,10,19,26]. In addition, these laminar patterns can be visualized utilizing magnetic resonance imaging, which holds great promise for clinical application [28]. Therefore, there is an imperative to better understand normal prenatal human brain development and alterations from it, as observed in diverse neurological and psychiatric disorders, such as autism, epilepsy, and schizophrenia.

To date, little is known about the spatiotemporal distribution of neuronal TFs and RBPs and differences in their laminar and regional organization in the human fetal brain. However, several studies [29,30,31,32] revived interest in early laminar cortical development and spatiotemporal distribution of different TFs and RBPs [13,24,25,33,34]. In particular, the role of deep projection neurons (DPN) during early prenatal neocortical development came under the spotlight recently, and their transcriptome profiles are associated with neurodevelopmental disorders, including autism spectrum disorders (ASD) [35,36].

The transition from embryonic to early fetal developmental period occurs by 8 PCW. This developmental transition is vital for the molecular specification and establishment of DPN identities together with Cajal–Retzius (CRN) and subplate neurons (SPN), both of which are neuronal subpopulations also associated with ASD [9,37]. DPN, CRN, and SPN neurons are among of the earliest-born neurons that eventually take different laminar positions in the neocortex: DPN is located between CRN and SPN, while the latter two acquire positions above and below the forming cortical plate (CP). During the early fetal period, DPN not only projects to subcortical centers in the basal ganglia, thalamus, brain stem, and spinal cord [38,39], but together with transient SPN and CRN neurons, inaugurates the primordial laminar-specific networks within the developing neocortex [27].

Early regional specification of laminas within the developing human neocortex has been described as the basic cortical subdivisions (“Grundgliederung”) (see review in [4] and [40]) and was recognized by classical studies [1,2,3,4,40,41]. The spatiotemporal differences in early postmigratory neuronal condensation into distinct laminas are noticeable at the time of CP formation (between 7.5 and 9 PCW) [5,7,26,42] and subplate (SP; between 8 and 15 PCW) formation. Thus, these differences represent a unique developmental period that might reveal regional differences in the molecular identities of postmigratory projection neurons. Despite the extensive research on the DPN origin, neuronal laminar identity, and molecular specification in lower mammals, numerous questions remain open in primates, especially during the early cortical development in humans. Overall, the molecular dynamics of laminar structures in regard to the human DPN, SPN, and CRN still remain largely unexplored.

To systematically analyze early DPN regional and laminar dynamics to disclose when and how highly ordered association cortex [43,44,45,46] displays the initial regional molecular patterning, we selected the anterior portion of the developing frontal lobe (prospective prefrontal cortex anlage). In primates, the frontal cortex projects to the subcortical structures involved in complex cognitive and behavioral functions [43]. Interestingly, the projections from the frontal subcortical associative cortex follow the timing of early motor cortex projection towards the subcortical centers, as previously described in the human fetal brain [47]. The revelation of developmental characteristics related to the human associative frontal cortex will serve as basic normative data for future studies of the abnormal changes caused by early cortical organization disturbances. Here, we utilized immunofluorescence (IF) coupled to cytoarchitectonics (Nissl) to complement the current large-scale molecular studies that are focused on the expression of hominini cortical type-specific gene networks [15,21,29,30,31,33].

The objective of this study was to gain insight into the postmigratory DPN, SPN, and CRN differentiation by analyzing spatiotemporal dynamics of the selected TFs and an RBP in the prefrontal cortex during early neocortical development. We also correlated these data with proliferative, synaptic, and fibrillar markers. A period between 7.5 and 15 PCW was selected as, during this time, the CP predominantly consists of DPN, and the SP undergoes a remarkable primate-specific expansion [48]. Here, we describe the laminar shifts and regional differences [13,49,50] of TFs (DPN markers: CTIP2, TLE4, SOX5, and SPN marker TBR1) [12,50,51,52] or an RBP CELF1 during five prenatal phases [5,6,7,53,54] of neocortical development: (1) late preplate (PP) at 7.5 PCW, (2) pioneering CP at 8 PCW, (3) first condensation of CP at 9 PCW, (4) SP formation at 13 PCW, and (5) typical fetal lamination at 15 PCW. By using this approach, we shed light on the common history of the CP layer VI and expanded SPN and solidified laminar and regional landmarks for future molecular and comparative clinical imaging studies.

## 2. Materials and Methods

### 2.1. Human Brain Tissue Processing

Experimental procedures were performed using postmortem human prenatal brain tissue from 7.5 PCW to 15 PCW. Tissue sampling was carried out in accordance with the regulations of the Declaration of Helsinki 2000. The postmortem histological tissue used in the research is part of the Zagreb Neuroembryological Collection and has ethical permission from the Internal Review Board of the Ethical Committee of the University of Zagreb School of Medicine. Postmortem human brains were immersion-fixed in 4% paraformaldehyde (PFA) in 0.1 M phosphate-buffered saline (PBS; pH = 7.4). The fetal age was determined based on the crown-rump length (CRL, in millimeters) and pregnancy records and expressed in PCWs. Tissue blocks were embedded in paraffin and sectioned on a microtome (Leica, SM2000R, Wetzlar, Germany) in a coronal plane on 10 μm sections. Part of the fetal material was provided by the Joint MRC/Wellcome Trust grant #099175/Z/12/Z Human Developmental Biology Resource. We have analyzed the frontal portion of the cerebral hemispheres containing a prospective developing frontal neocortex. Analyzed frontal regions were selected from the mid-lateral and mid-dorsal parts of hemispheres above the level of the rostral portion of ganglionic eminence, above the pallial–subpallial border. The majority of section planes were coronal, eventhough semi-horizontal sections were also analyzed. The most rostral (anterior) polar portions were excluded from the analysis since they included tangential section planes. We have analyzed two–five specimens from each developmental phase. Notably, we have analyzed two specimens from the late preplate phase (7.5 PCW). One of them was well preserved and embedded in epon-araldit for electron microscopical procedure, serially cut in 1 micron thick plastic sections. Another specimen, fixed in 4% PFA and embedded in paraffin, was processed with immunofluorescence.

### 2.2. Histological and Immunofluorescence (IF) Stainings

Sections were stained using classical histological Cresyl violet (Nissl) staining to get a cytoarchitectonic overview. Immunofluorescence stainings were performed following a standard protocol: foremost, slides were immersed in Xylol solution with two changes for 10 min for the deparaffinization process; than to rehydrate sections, descending series of alcohols (100% EtOH (2 × 5 min), 96% EtOH (2 × 5 min), and finally, 70% EtOH (1 × 5 min) were used, following a rinsing in 1× PBS buffer. Antigen retrieval was performed by boiling sections in a citrate buffer, pH = 6.0. A blocking solution (1% BSA, 0.5% TRITON X-100 in 1× PBS) was applied for 2 h at room temperature (RT). Primary antibodies (Table 1) were incubated overnight in a humid chamber at +4 °C. The next day slides were washed in 1× PBS buffer (3 × 10 min), secondary antibodies (Table 1) were incubated for 2 h at RT in the dark, humid chamber, followed by washes in 1× PBS (3 × 10 min). Finally, tissue sections were treated with TrueBlack^®^ Lipofuscin Autofluorescence Quencher solution (Biotium, Fremont, CA, USA) for 30–60 min to prevent autofluorescence, washed with 1× PBS. Finally, sections were mounted using a mounting medium with DAPI (Vectashield^®^) and coverslipped.

### 2.3. Immunochemicals and Reagents

A list of primary and secondary antibodies used in this study to identify molecular characteristics of cells and surrounding structures is shown in Table 1.

### 2.4. Imaging

Analysis was done by obtaining images of the lateral, dorsal, medial, and basal portions of the developing frontal lobe on the coronal sections (note that section level is shown by schematic presentation in the upper left corner of each figure). Images were taken by a high-resolution slide scanner Hamamatsu NanoZoomer 2.0 RS system using a 40× (NA 0.75) objective lens at 455 nm/pixel resolution. Fluorescence images were taken with the Hamamatsu LX2000 Lightning exciter, in addition to a confocal laser scanning microscope Olympus FV3000 with a 20× objective (UPlanSApo, NA 0.75, Olympus) using FV31S-SW Fluoview software at a resolution of 1024 × 1024 pixels. All images were acquired with two channels (488 and 561 laser lines) and figures assembled in Microsoft Publisher.

### 2.5. Image Processing and Statistical Analysis

Cell counting procedure was performed utilizing ImageJ (FIJI) software, following established protocols [55,56] by using Otsu’s thresholding criteria for the 8-bit images. ROI for each image was determined by using the same shape and size parameters across all samples analyzed. Moreover, the particle-analysis plugin in ImageJ was used with the following parameters: size: 1-infinity; circularity: 0.00–1.00. The total number of positive cells present in each previously determined ROI for each image was graphically presented in the results. IF-stained coronal sections depicting dorsal and basal portions of the human developing frontal lobe (section level can be seen on schematic presentation in the upper-left corner of the figure) were delineated into ten bins corresponding to the actual cortical compartments, determined on the adjacent Nissl section. Cells were counted in ten bins on the dorsal and basal portions of the frontal lobe and graphically presented as a percentage of DPN marker-positive cells in the cortical compartments.

## 3. Results

Here we describe the laminar shifts and regional differences [13,49,50] of several TFs used either as DPN (CTIP2, TLE4, SOX5) or SPN (TBR1) markers [12,50,51,52] and the RBP CELF1 during five prenatal phases [5,6,7,53,54] of neocortical development, namely: (1) late preplate (PP) at 7.5 PCW, (2) pioneering CP at 8 PCW, (3) first condensation of CP at 9 PCW, (4) SP formation at 13 PCW, and (5) a typical fetal lamination at 15 PCW.

**Figure 1:**
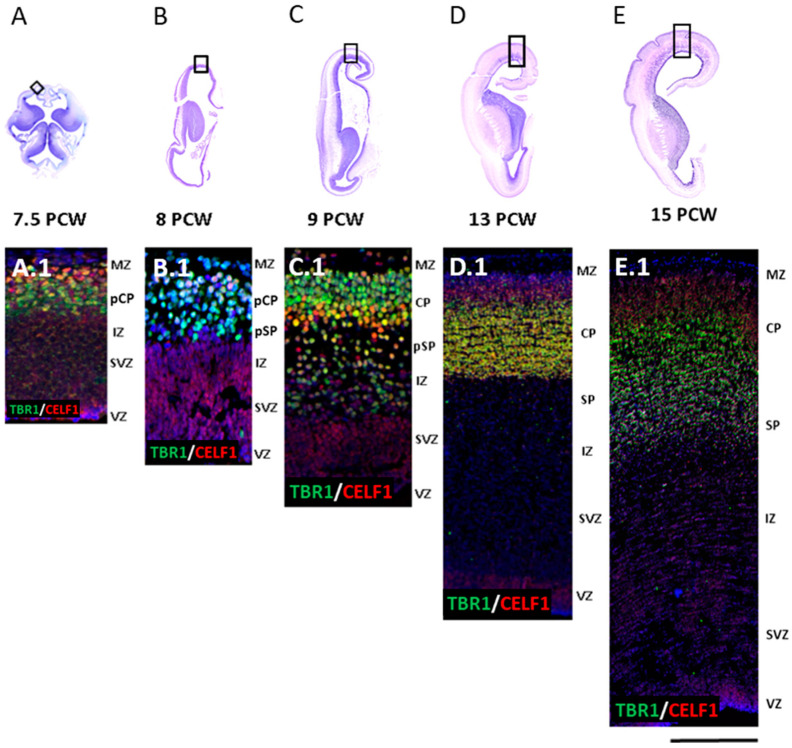
Panoramic view of the TF TBR1 and RBP CELF1 expression in early DPN and SPN neurons of the prospective frontal cortex during five initial phases of fetal development (7.5 PCW and 15 PCW). (**A**–**E**) Nissl-stained sections show developmental changes in morphology and size increase of the human brain from 7.5 to 15 PCW. Boxes depict position where corresponding and representative confocal (**A.1**–**E.1**) images were taken from sections immunostained for TF TBR1 (green) and RBP CELF1 (red). Well-established SPN marker, TBR1, is mainly expressed in the pioneering cortical plate (pCP; (**A.1**,**B.1**)) starting from 7.5 PCW. In addition, TBR1 is expressed in the presubplate (pSP; (**B.1**,**C.1**)) between 7.5–9 PCW, while at later phases (9–15 PCW), TBR1 is expressed in the CP, pSP, and SP (**C.1**–**E.1**). The RBP CELF1 is expressed in early proliferative zones, the ventricular zone (VZ) and subventricular zone (SVZ), as well as in the CP neurons. Colocalization between TBR1 (green) and CELF1 (red) was found mostly in CP (yellow). DAPI is shown in blue. Scale bar = 400 μm.

Systematic analysis of IF-processed coronal sections (compartmental approach) showed an early laminar distribution of TFs TBR1 and RBP CELF1 in DPN in all compartments during the early- and mid-fetal development (from 7.5–15 PCW) of the human frontal lobe, best revealed in panoramic presentation (Figure 1) and discussed as early basic frontal regionalization in the first part of the discussion.

### 3.1. Late Preplate Phase (7.5 PCW)

**Figure 2:**
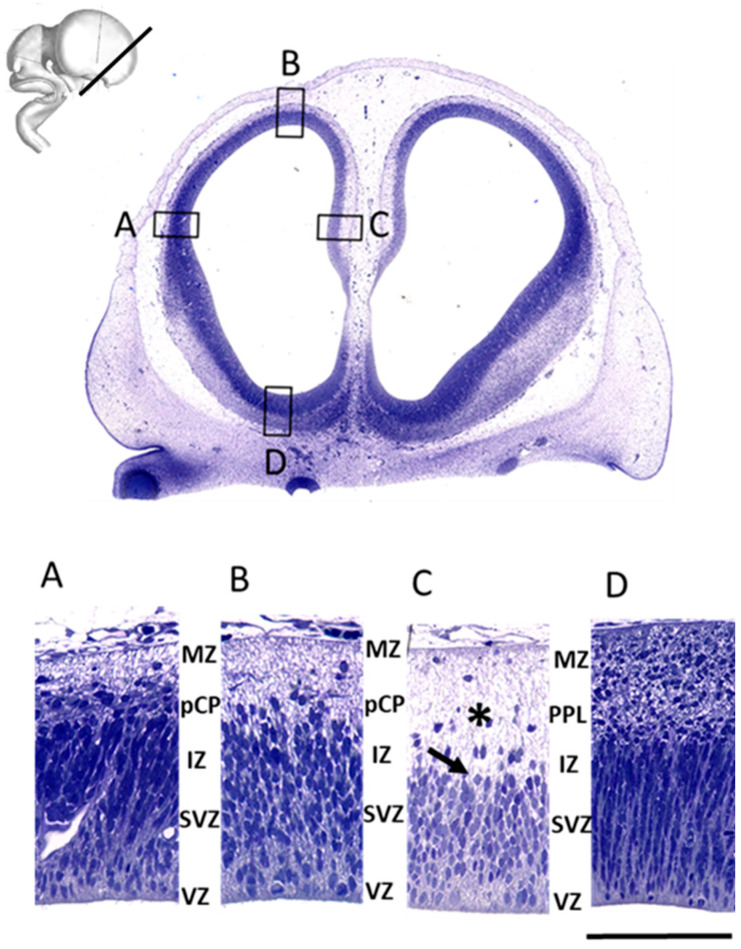
The frontal portion of cerebral vesicles shows cytoarchitectonic regional differences in the developing neocortices in the late preplate phase at 7.5 PCW. (Top) Nissl-stained coronal section of the frontal neocortex. Boxes depict enlarged images shown in (**A**–**D**). (**A**–**D**) The earliest transient fetal zones are seen on a 1-micron thick Nissl-stained section: proliferative ventricular zone (VZ), wider and more cell-dense subventricular zone (SVZ), intermediate (IZ), late preplate zone (PPL) with first aggregated cells on the medial (**C**) part, cell dense pioneering cortical plate (pCP) on lateral (**A**), dorsal (**B**) and basal (**D**) part, and the most superficial marginal zone (MZ). The less cellular late-preplate sublayer is marked with an asterisk, while the deep sublayer with more dense cellularity is marked with an arrow. Scale bar = 200 μm.

In the earliest examined specimen at 7.5 PCW (Carnegie Stage 22), lateral cerebral wall is composed of the following laminas starting from lateral ventricles: ventricular zone (VZ), subventricular zone (SVZ), intermediate/preplate zone, and the marginal velum (“Randschleier”) [9]. Given that the border between the fibrillar intermediate zone (IZ) and cellular preplate (PP) cannot be precisely defined even on 1-micron thin sections (Figure 2), we described it as the “intermediate/preplate compartment or a mantle”. Importantly, the intermediate/preplate zone shows early distinct sublaminar and regional organization. The most superficial (adpial) sublamina shows less cellularity and the presence of large scattered cell bodies (Figure 2, asterisk), whereas the deep sublayer, including the fibrillar part of developing IZ, shows more cellularity (Figure 2, arrow). In addition, there are prominent regional differences in the thickness of the intermediate/preplate zone between the basal and dorsal parts of the cerebral wall. In the basal portion, the intermediate/preplate zone is much thicker, with the tendency to narrow down towards the dorso-rostral regions of the cerebral vesicle.

**Figure 3:**
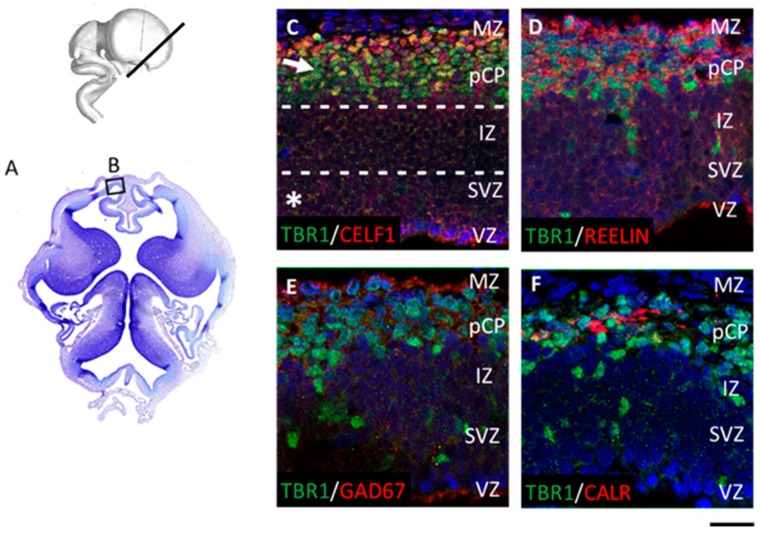
Main compartments found on a semi-horizontal section at 7.5 PCW. (**A**) The Nissl-stained semi-horizontal section at 7.5 PCW. Box (**B**) denotes the prospective place where representative confocal images were taken in (**C**–**F**). (**C**–**F**) The proliferative compartment ((**C**), asterisk) is present below the intermediate zone (IZ). Developing pioneering cortical plate (pCP) represents a newly-formed CP compartment, which contains postmigratory neurons ((**C**), arrow) above the IZ. Representative confocal images of immunostained sections (**C**–**F**). At 7.5 PCW, clusters of radially aligned TBR1-positive (+) neurons were found to reach early pCP and marginal zone (MZ), suggesting the presence of radial migration event from the proliferative compartment towards pCP. Late preplate/pCP shows sublaminar organization (**D**): REELIN+/TBR1+ CRN at the superficial part and REELIN-/TBR1+ cells aligned at the deeper part. CELF1, used as the glutamatergic neuron marker, colocalized with the DPN marker TBR1 (**C**), whereas GABAergic GAD67+ (**E**) and calretinin (CALR)+ cells (**F**) did not colocalize with TBR1+ neurons. Scale bar = 50 μm.

Furthermore, at the late preplate phase, two essential cerebral wall compartments are established. The proliferative compartment (Figure 3C, asterisk) is present before the fibers divide main proliferative zones VZ and SVZ into inner SVZ (iSVZ), inner fibrillar layer (IFL), and outer SVZ (oSVZ). Another compartment (Figure 3C, arrow) is formed as a site of maturation and molecular specification of postmigratory neurons. These neurons differentiate into three subpopulations: future SPN [TBR1-positive (+)], future layer VI neurons (DPN markers+) (Figure 3C), and CRN (REELIN+) (Figure 3D). Between these two large compartments, a migratory zone is settled with diverse neuronal markers profile. TF TBR1+ neurons colocalize with the RBP CELF1 in deep CP and the pCP (Figure 3C). In addition, in the MZ, TBR1 colocalizes with REELIN, which characterizes CRN (Figure 3D), but not with GABAergic markers GAD67 (Figure 3E) or CALR (Figure 3F).

### 3.2. Initial Formation of Pioneering CP Phase (8 PCW, Carnegie Stage 23)

**Figure 4:**
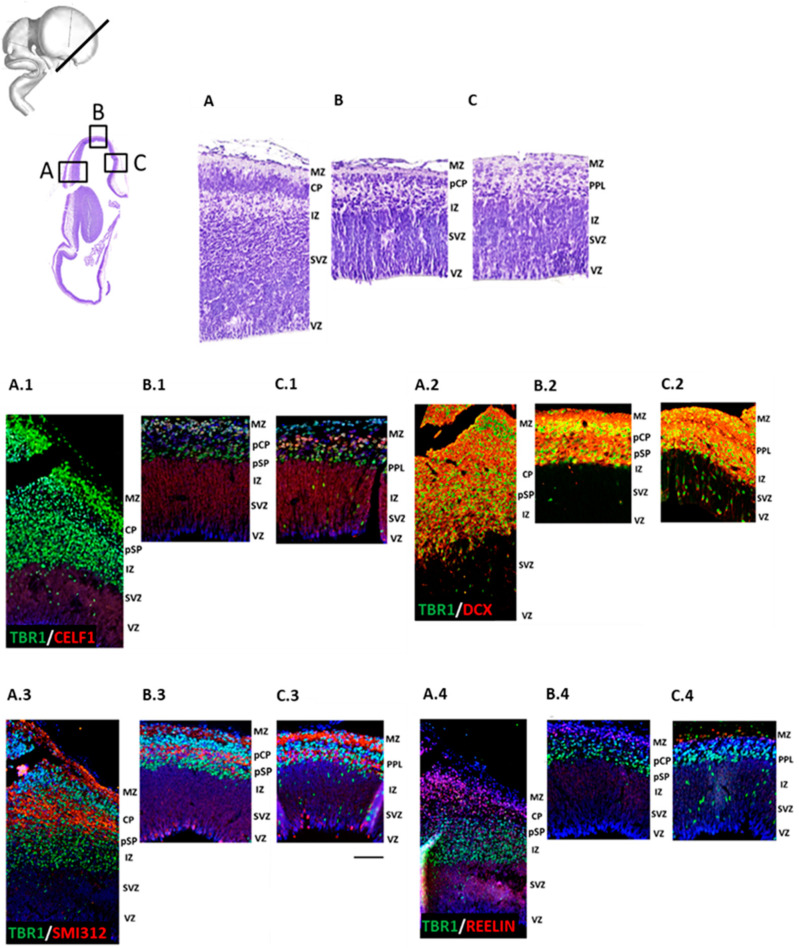
Early regional differences in the frontal lobe appear during the initial formation of pioneering CP at 8 PCW from lateral to dorsal and medial regions. (**A**–**C**) Regional cytoarchitectonic differences in the frontal lobe can be detected as early as 8 PCW on representative Nissl (**A**–**C**) and adjacent IF confocal images (**A.1**–**C.4**). The late preplate (PPL) and first cortical columns are seen in the medial portion of the frontal lobe. In contrast, the pioneering cortical plate (pCP) is found in the dorsal portion of the developing frontal neocortical wall. The lateral cortical portion is characterized by the initial CP formation. CELF1 is expressed in proliferative zones (**A.1**–**C.1**) in the late preplate cells (**C.1**) and upper part of pioneering cortical plate cells (**B.1**), where it colocalizes with TBR1. TBR1+/DCX+ (**A.2**–**C.2**) neurons migrate towards CP to reach their final position. REELIN+ CRN is present in the MZ (**A.4**–**C.4**) and the upper part of the initial CP (**A.4**). Fibrillar marker SMI312 shows the first axonal fibers stretching through the CP and the MZ (**A.3**–**C.3**). ((**A**), (**A.1**–**A.4**)—lateral, (**B**), (**B.1**–**B.4**)—dorsal, and (**C**), (**C.1**–**C.4**)—medial portion of the developing frontal cortex). Scale bar = 100 μm.

The formation of the “primitive pioneering CP” (pCP) marks the transition from the embryonic to the fetal developmental period when the first form of CP starts to condense from lateral to dorsal and medial aspects of the frontal pallium. We thus analyzed lateral (Figure 4(A.1)), dorsal (Figure 4(B.4)), and medial (Figure 4(C.4)) portions of the developing frontal cortex on the sections semi-horizontally cut. In the prospective frontal lobe at 8 PCW, we found regional lateral to medio-dorsal differences at both cytoarchitectonic levels and after analyzing DPN markers distribution (Figure 4). The initial formation of CP occurs in the lateral portion of the developing frontal cortex (Figure 4A), as characterized by the expression of TF TBR1 (Figure 4(A.1)–(A.4)). Pioneering cortical plate (pCP) can be detected in the dorsal portion of the developing frontal cortex (Figure 4B), showing radially-oriented columns of early neurons expressing both TBR1 (Figure 4(B.1)–(B.4)) and RBP CELF1 (Figure 4(A.1)). The late preplate (PPL) organization is noted in the medial portion of the developing frontal cortex (Figure 4C), which shows sparsed cells with a simple organization. Late PPL cells are mostly TBR1+ (Figure 4(C.1)–(C.4)) and CELF1+ (Figure 4(C.1)). Furthermore, REELIN+ CRN+ neurons in the most superficial compartment, i.e., MZ (Figure 4(A.4)–(C.4)), show the most extensive expression pattern in the lateral portion (Figure 4(A.4)). An extensive (DCX+) migratory zone (Figure 4(A.2)–(C.2)) that enables immature neurons to reach their targeted destination displays intense migration from the VZ and SVZ in the medial portion (Figure 4(C.2)) of the developing frontal neocortex. Finally, we detected early axonal fibers using the fibrillar marker SMI312 and marked two axonal tracts stretching through the CP and the MZ (Figure 4(A.3)–(C.3)). These data further confirm early molecular divergence of the developing frontal neocortical wall that is described in the first section of the discussion.

### 3.3. First Condensation of the CP Phase (9 PCW)

**Figure 5:**
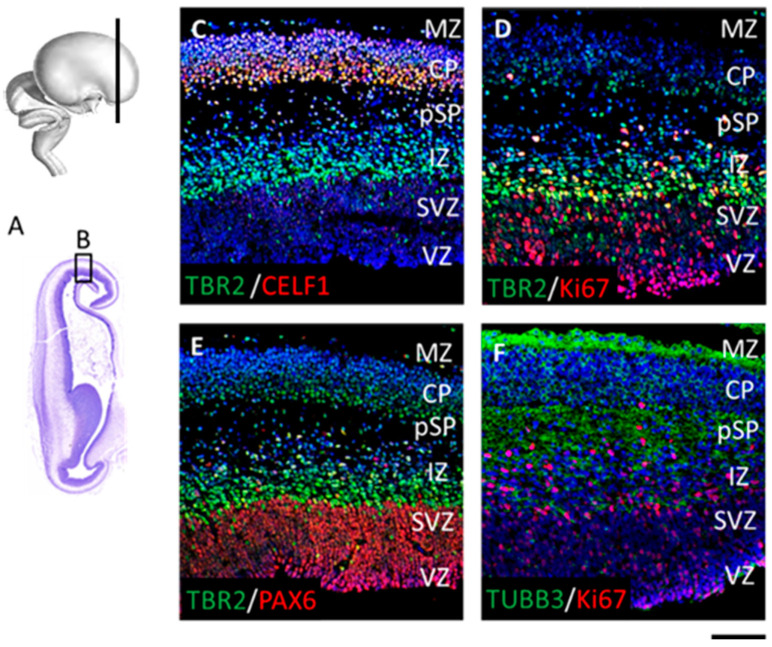
The prospective frontal lobe shows the first CP condensation lateral-to-medial differences at 9 PCW. Dorsolateral portion (**B**) of the Nissl-stained coronal section (**A**) is represented by magnified IF-stained adjacent sections (**C**–**F**). Note radially densely aligned cells in the CP. Proliferative Ki67+ cells (**D**) and progenitor PAX6+ cells (**E**) are positive in the VZ and SVZ. Intermediate progenitor (IP) TBR2+ cells (**C**–**E**) are predominantly located in the SVZ and the IZ. The CELF1+ future projection neurons (**C**) occupy the CP and colocalize with TBR2+ cells in the deep part of the CP. Note that migratory neurons display oval elongated soma (**C**–**E**). TUBB3 is found in neuronal microtubules, outlining cell bodies, immature axons, and dendrites (**F**). Scale bar = 100 μm.

**Figure 6:**
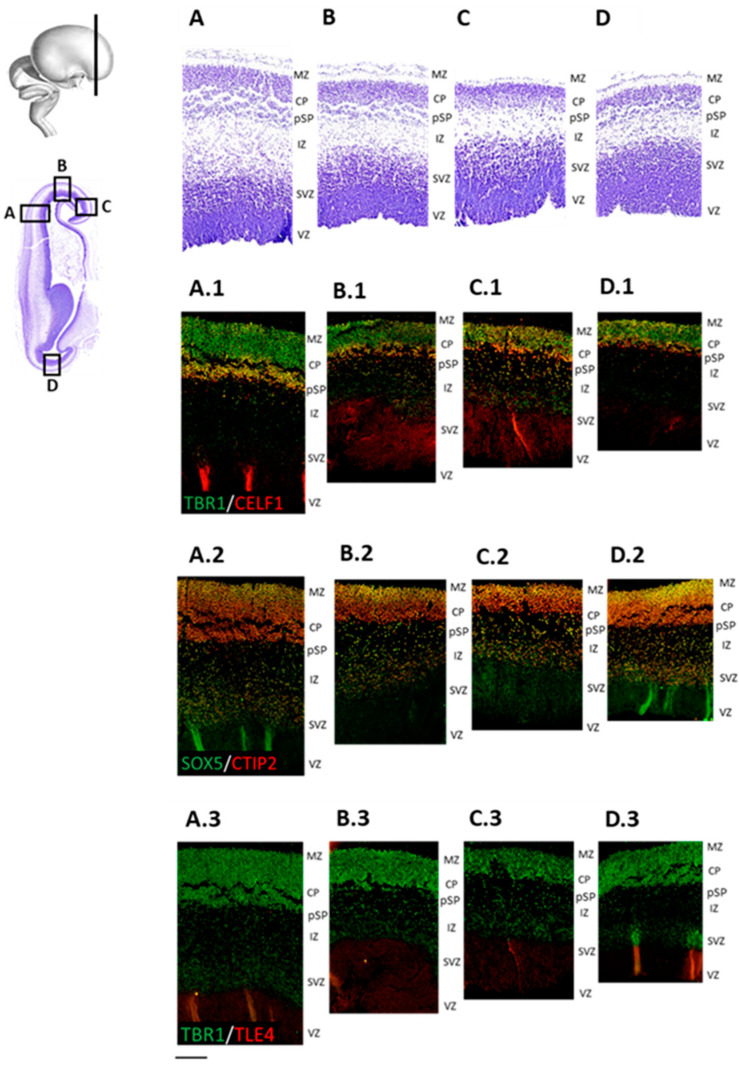
Regional differences in cytoarchitectonics and DPN expression dynamics at 9 PCW. (**A**–**D**) Nissl-stained coronal sections show enlarged regional portions of the early-fetal frontal lobe. Superficial CP can be distinguished from the deep portion of the CP at the phase of the first CP condensation (9 PCW). Boxes depict areas of adjacent immunostained sections enlarged in (**A1**) to (**D3**). Representative confocal images reveal regional differences in the DPN markers expression. TBR1 (**A.1**–**D.1** and **A.3**–**D.3**) is noticed in all parts of the CP and well-developed proliferative SVZ. Projection neuron markers RBP CELF1, as well as TFs SOX5 and CTIP2, are expressed in the early CP. CELF1 (**A.1**–**D.1**) shows stronger reactivity in the deeper CP, where it colocalizes with TBR1. SOX5 and CTIP2 (**A.2**–**D.2**) are equally distributed through the first condensed CP. Future DPN marker TLE4 (**A.3**–**D.3**) is still not expressed by 9 PCW. ((**A**), (**A.1**–**A.3**)—lateral, (**B**), (**B.1**–**B.3**)—dorsal, (**C**), (**C.1**–**C.3**)—medial, and (**D**), (**D.1**–**D.3**)—basal portion of the frontal lobe). Scale bar = 200 μm.

**Figure 7:**
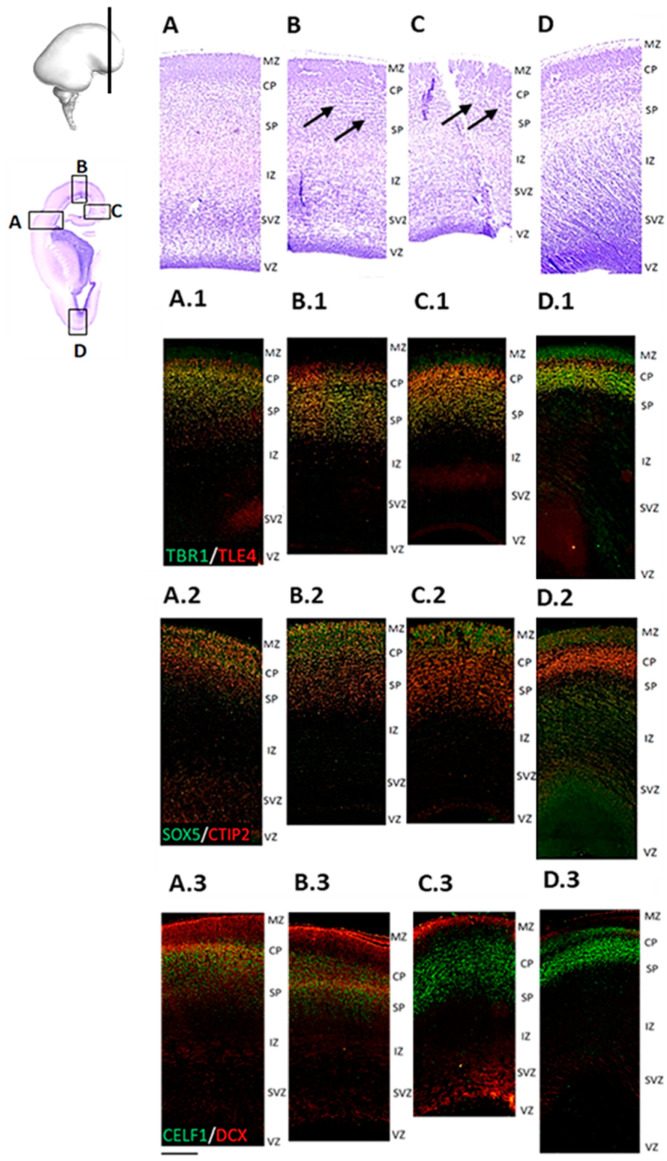
Subplate formation in the frontal cortex is a characteristic feature of cortical development during 13 PCW. Regional differences are visualized on the enlarged portions of the Nissl-stained section (**A**–**D**) and adjacent IF stainings (**A.1**–**D.3**). Waves of migratory neurons in the CP can be discerned in the medial and dorsal parts of the developing frontal cortex. ((**B**,**C**) arrows). Note that the most superficial part of CP is lacking TBR1 expression (**A.1**–**D.1**). CELF1 is expressed in the CP, the SP, as well as in VZ and SVZ. DCX signal shows migratory cells from VZ to the CP. Detected DCX immunopositivity above the CP could suggest diverse DCX developmental roles (**A.3**–**D.3**). CTIP2 shows an occasional colocalization with SOX5, the latter which is more expressed in the upper part of the CP. CTIP2 signal is more intense in the lower CP and the SP (**A.2**–**D.2**). ((**A**), (**A.1**–**A.3**)—lateral, (**B**), (**B.1**–**B.3**)—dorsal, (**C**), (**C.1**–**C.3**)—medial, and (**D**), (**D.1**–**D.3**)—basal portion of the developing frontal cortex). Scale bar = 200 μm.

At 9 PCW, early radially-oriented columns in a newly-forming CP are condensing, thus contributing to the establishment of CP. This process is necessary for the development of the ultimate six-layer neocortex during the later phases. Our results show cytoarchitectonic condensation of the CP on Nissl-stained sections, as well as DPN marker distribution in the CP on the adjacent IF-stained sections (Figure 5 and Figure 6). To analyse the cell proliferation, maturation, and specification in different neocortical regions, we used double-labeling IF techniques (Figure 5). As expected, two main proliferative zones, VZ and SVZ, show the presence of both Ki67+ and PAX6+ progenitor radial glia cells (Figure 5E,F), while intermediate progenitor TBR2+ cells mainly occupy SVZ and IZ. TBR2+ positive cells are also present in the deep part of the CP (Figure 5C–E), along with CELF1, expressed in the first condensed CP (Figure 5A). According to TUBB3 expression, the postmigratory neuronal cell bodies did not yet acquire the mature pyramidal shape at 9 PCW but displayed immature axonal and dendritic neurite extensions (Figure 5F). The SP marker TBR1 is predominantly expressed in the first condensed CP and pSP (Figure 6(A.1)–(D.1) and Figure 7(A.3)–(D.3)), where it colocalized with RBP CELF1 (Figure 6(A.1)–(D.1)). Additional DPN markers, SOX5 and CTIP2 (Figure 6(A.2)–(D.2)) are colocalizing in the superficial portion of the CP. Furthermore, SOX5 and CTIP2 expression patterns are detected in the pSP and migratory IZ. These data suggest stronger lateral-to-dorsal-to-medial differences in the developing frontal human neocortex at 9 PCW.

### 3.4. Formation of the Subplate Phase (13 PCW)

**Figure 8:**
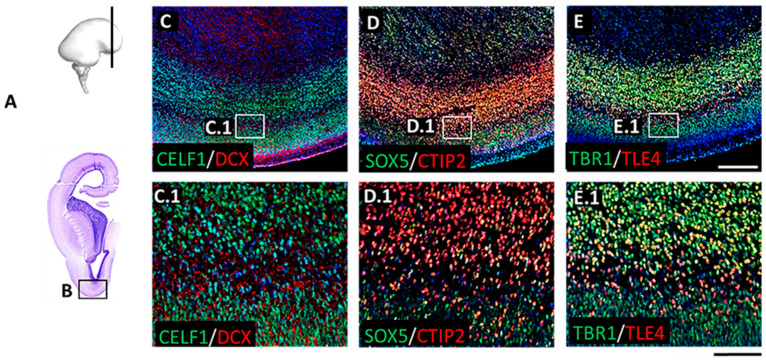
The human frontal lobe shows a “double plate” pattern as a unique feature of the orbitobasal cortex during the subplate formation phase at 13 PCW. Magnified portion (**B**) of the Nissl-stained section (**A**) displays orbitobasal cortex represented on IF-stained subsequent sections (**C**–**E**), coupled with corresponding magnified parts (**C.1**–**E.1**) showing different projection neuron markers. In this frontal region, there is no gradual loosing of deep CP but a dense accumulation of DPN markers CELF1 (**C.1**), CTIP2 (**D.1**), SOX5 (**D.1**), TLE4 (**E.1**), and SPN marker, TBR1 (**E.1**), reactive nuclei giving a unique “double-plate”-pattern appearance. Cell-sparse layer in between “two plates” is DCX+ (**C.1**), pointing to the cell migration towards the upper levels. DPN markers are also expressed in the sparsed cells of the intermediate layer (**C.1**–**E.1**). Scale bar (**C**–**E**) = 200 μm. Scale bar (**C.1**–**E.1**) = 100 μm.

**Figure 9:**
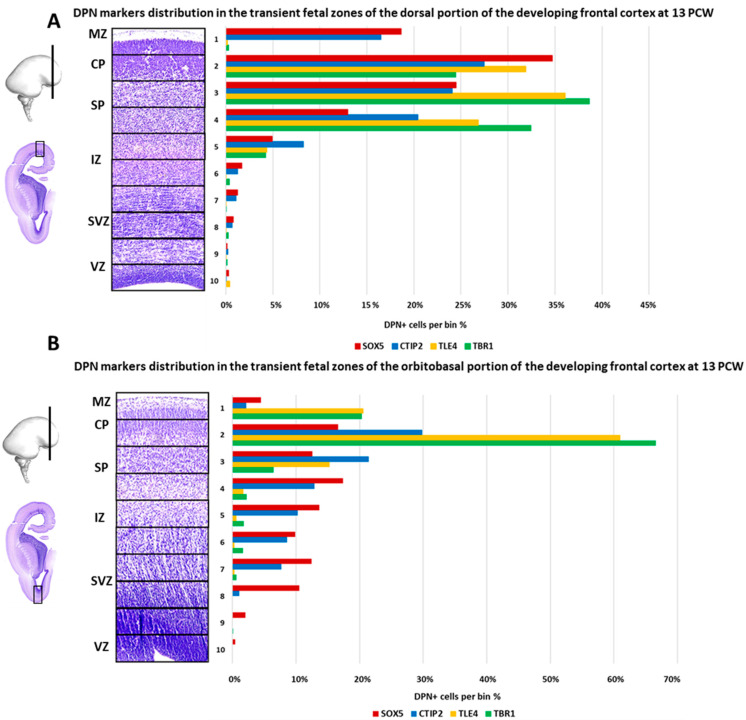
Laminar distribution of DPN markers in the dorsal and basal portion of the frontal cortex in the developing human brain during the SP formation period at 13 PCW. Cells were counted on the IF-stained dorsal (**A**) and basal (**B**) portions of the frontal cortex (coronal sections). The quantification columns were divided into ten bins, delineated according to the adjacent Nissl sections. Quantitative data obtained by cell counting is presented as a percentage of DPN marker (SOX5, CTIP2, TLE4, TBR1) positive cells in the cortical compartments. DPN markers are predominantly expressed in bins 2, 3, and 4, corresponding to the deep CP and the SP compartments.

The prominent laminar changes in the human neocortex occur at 13 PCW that are mainly related to the formation of the SP. DCX immunostaining revealed waves of migratory neurons destined to reach their final laminar position. In addition, we found a distinct distribution of DPN markers (Figure 7) characterized by the expression of RBP CELF1 (C1), TFs CTIP2 (D1), SOX5 (D1), and TLE4 (E1), as well as SPN marker, TBR1 (E1), in different regions of the developing frontal human neocortex (Figure 7(A.3) and Figure 8(D.3)). Moreover, we discovered a „double plate“ feature, which is unique to the orbitobasal region of the frontal cortex (Figure 8). Cell quantification showed that the distribution pattern of DPN markers in the dorsal portion of the developing frontal cortex tends to be more uniform. In addition, the majority of counted cells were evenly accumulated in the upper transient fetal zones (including CP and SP) when compared to the orbitobasal portion of the frontal cortex. The distribution of DPN markers in the orbitobasal cortex showed the highest percentage of TBR1 and TLE4 immunoreactive cells in the deeper portions of the CP, in contrast to the CTIP2 and SOX5 immunoreactive cells (Figure 9). Further elaboration of our novel data showing these unique features of the developing prospective frontal lobe is described in more detail in a second section of the discussion. Collectively, our results suggest significant differences in the DPN distribution pattern within transient fetal zones between the dorsal and basal portions of the developing frontal cortex.

### 3.5. Typical Fetal Lamination Phase (15 PCW)

**Figure 10:**
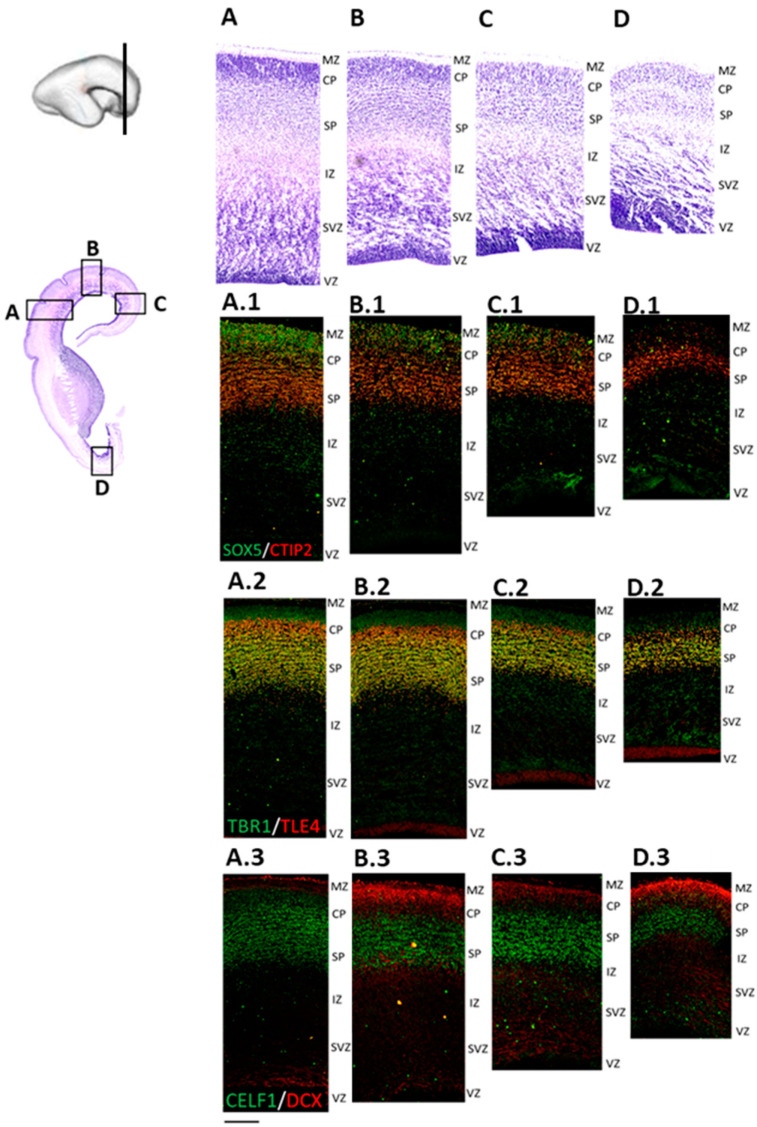
Establishment of the typical fetal lamination during the mid-fetal period (15 PCW). Magnified regional portions of the Nissl coronal section show a cytoarchitectonic overview of the typical fetal lamination (**A**–**D**). IF-stained sections show regional and laminar dynamics of DPN markers expression (**A.1**–**D.3**). Expanded SP shows TBR1 reactivity throughout the whole thickness (**A.2**–**D.2**). SPN are labeled with deep-projection neuron markers TLE4 (**A.2**–**D.2**), SOX5, and CTIP2 (**A.1**–**D.1**), as well as CELF1 (**A.3**–**D.3**). Note that SOX5 shows the strongest signal in the most superficial part of the CP. CTIP2 shows a strong signal in the lower CP and the SP. TLE4 is expressed in the lower CP and SP. In addition, DCX positive signal marks migratory zones from VZ to CP. Layer IV and III neurons are already born during the mid-fetal period. ((**A**), (**A.1**–**A.3**)—lateral, (**B**), (**B.1**–**B.3**)—dorsal, (**C**), (**C.1**–**C.3**)—medial, and (**D**), (**D.1**–**D.3**)—basal portion of the frontal cortex). Scale bar = 200 μm.

All “typical” transient fetal compartments can be easily distinguished on the frontal cortex at 15 PCW: proliferative zones VZ and SVZ, migratory zones IZ and SP, differentiating compartments CP and SP, and the most superficial MZ. Our results (Figure 10(A.1)–(D.3)) suggest the existence of the differential regional expression of the DPN marker during the mid-fetal period and point to the size differences in the transient fetal zones within the developing frontal lobe (Figure 10A–D). The DPN markers CTIP2 and SOX5 (Figure 10(A.1)–(D.1)) were found in the CP and the SP; their colocalization is observed mostly in the superficial portions of the CP. The SOX5 expression was more prominent in the superficial parts of the CP that gradually diminished in the latero-medial gradient and the latero-basal portion of the developing prospective frontal cortex (Figure 10(A.1)–(D.1)). Double-labeling between SPN marker TBR1 and DPN marker TLE4 (Figure 10(A.2)–(D.2)) was found in both the CP and the SP. Whereas TLE4 was predominantly expressed in the superficial part of the CP, TBR1 was expressed in the SP and deeper parts of the CP. RBP CELF1 was expressed in the CP and the SP throughout the entire developing frontal cortex and in the SVZ of the basal portion of the frontal cortex (Figure 10(A.3)–(D.3)). Migratory marker DCX was present through the whole cortical thickness with the highest intensity in the MZ, the VZ, and the SVZ (Figure 10(A.3)–(D.3)). These new data present dynamics of early components in transient compartments that differentiate during the early fetal period; they are further discussed in the third section of the discussion. This developmental phase is characterized by the most pronounced regional differences in TFs and an RBPs expression pattern in the developing neocortex.

## 4. Discussion

In this study, as expected, we have found that the first condensed CP at 8 PCW consists of DPN. When analyzed in different regions (the dorsolateral, orbitobasal, and medial prospective prefrontal cortex), DPN showed region-specific expression patterns and organization differences identified several months before final neocortical arealization. Analysis of the entire cortical cellular compartments (compartmental approach) during the SP formation (expansion) phase (at 12–14 PCW) shows novel aspects of regional molecular geography. Co-expression of proliferative (PAX6+, Ki67+) and DPN markers (TBR1+) revealed unique frontal cortex cytoarchitectonic features and outlines of well-developed outer SVZ (oSVZ). Contrary to the general view, we found that basic neuronal network components of the developing prefrontal cortex differentiate early in fetal life, concomitantly with regional and molecular differentiation of the central nervous system. This high-order associative neocortical region thus differentiates one to two weeks after primary motor [57] and sensory cortical regions [5]. These three novel aspects of our results are further discussed in the following sections.

### 4.1. The DPN Role in the Early Basic Frontal Cortex Regionalization and Architecture Establishment

Previous seminal experimental studies and human postmortem material analyses showed that early regional differentiation begins with the gradient expression of different TFs or area-patterning genes [17,32,51,58,59,60], leading to the primordial area map (protomap) formation [23,60,61,62,63,64]. The final areal formation is achieved by the interplay of these intrinsic determinants with different extrinsic factors, especially with thalamic input and instruction [58,59,60,61,65,66]. Laminar organization of proliferative, migratory, and post-migratory embryonic compartments [6,9,19,61,67,68] occurs in parallel with the establishment of molecular and signaling gradients. An early laminar pattern that outlines the major cortical divisions (isocortex, mesocortex, archicortex, and paleocortex) was thoroughly studied in the classical literature [1,2,3,4,40,41] and is described as basic histogenetic cortical subdivisions [4]. We have observed radially aligned cells and radial nuclear aggregations corresponding to the embryonic columns described by [62] and [69]. According to the radial unit hypothesis [62,64], cytoarchitectonically-distinct early embryonic columns of the CP are formed by postmigratory neurons originating in the proliferative zones, which then migrate along the radial glia fibers into differentiating zones. It is still under debate whether embryonic columns correlate with the adult columnar cortical organization [70]. The potential functional significance of embryonic columns is that early CP cells exchange signaling via electrical junctions and contribute to the early oscillatory neocortical activity [37,66]. Thus, early cell-to-cell communication within the embryonic columns may influence further neuronal network development and maturation in the cerebral cortex [66,70].

The results of our study are in accordance with the so-called unifying theory of Marin Padilla [71], who proposed that the ontogenesis of pyramidal cells in the mammalian neocortex is a hallmark of developmental cytoarchitectonics. Namely, DPN, which shows TBR1 reactivity and resides in the dense part of early CP [20,69,71], belongs to pyramidal neurons which are the backbone of cortical organization. In other words, early “condensed CP” before 12 PCW is actually a transient compartment composed of the first-born (“older”) deep pyramidal neurons, which correspond to densely packed TBR1 reactive nuclei revealed in our IF-stained sections. The concept of pyramidal neurons as basic elements in the early cortex in this paper is corroborated by the appearance and distribution of other neuronal elements, such as SPN and CRN, as the transient population of projection neurons. However, our study was partly limited by the availability and properties of the analyzed postmortem human tissue material. Further elaboration requires additional molecular and genetic studies and experimental analysis.

Our results of the DPN laminar dynamics within the fetal compartments relate to the period of regionalization that was not frequently in the focus of the previous studies [5]: spanning from the preplate phase to the mid-fetal typical lamination with pronounced SP. The first Brodmann maps and basic six-layered lamination [72] develop much later than the fetal stage, as reported for the human preterm cortex [3,4,28,73,74]. During the initial developmental phases, lamination patterning proceeds from lateral towards the dorsal and then to the medial portion of the anterior frontal cortex, showing the same mode of the sublaminar organization as observed in other parts of the isocortex. Our results regarding the compartmentalized distribution of DPN markers serve as an important criterion for regional patterning at a time when CP is not yet fully laminated [28]. The mode of lamination formation is ubiquitous, but the tempo, timing, and extent of observed DPN molecular dynamics show regional differences. Thus, DPN TFs and RBP CELF1 used in this study can serve as reliable regional maturation markers. Notably, we have found that DPN establishes regional differences between lateral orbitobasal and medial frontal cortex long before an intersynaptic interaction with layer IV thalamic afferents [28,42] and supragranular layers differentiation occurs.

The distribution, packing density, and CP thickness marked with TBR1+ nuclei showed several unique frontal cortex characteristics that are not observed in other parts of the developing cerebral cortex. First, the unique characteristic is visible on the orbitobasal frontal cortex where DPN aggregate below the cell-dense CP, forming another cell-dense layer that resembles a second cortical plate (“double plate”). This intriguing phenomenon became prominent around 13 PCW during the SP formation phase. Other features that we found to be specific to the prefrontal cortex are rows of neurons with TBR1+ nuclei separated by cell-poor fibrillar strands. This phenomenon was observed in the medial, dorsal, and basal developing frontal cortex. We interpret this as characteristic semi-tangential fibers originating from the sagittal strata [75] and migratory neuronal waves produced in the proliferative SVZ [76,77]. Finally, SP is poorly delineated from the CP (above) and external capsule of IZ (below), indicating higher cellularity of this compartment compared to other cortical regions. This phenomenon changes later due to the increase in fibrilar and extracellular components of the voluminous SP [78].

The early involvement of TBR1+ DPN and SP neurons in regional differentiation and connectivity establishment is in accordance with reported early DPN gene expression network studies [15,30,31] in rodents [14,51,52] and humans [6,68]. This pivotal event occurs between the late preplate-pioneering plate phase [6,53,79], including the first condensation of the CP phase [42,54,80], when radially-oriented prospective migratory TBR1+ nuclei are detected. The radially migrating DPN originate most likely from the SVZ where TBR1+ and TBR2+ nuclei coexist along with a proliferative marker Ki67, possibly shedding new light on the SVZ role in the human brain.

### 4.2. DPN Distribution during SP Formation-Expansion Phase and Establishment of the Regional Frontal Cortex Geography

Our results have shown that DPN TFs markers are excellent indicators of the events related to the SP formation (expansion) phase in the human frontal cortex. The mode of SP formation by secondary expansion was previously described by structural criteria [5]. Cell dynamics by autoradiographic labeling of the expanded SP compartment were also reported for the first time in experimental primates by [48]. In the present study, we have shown that analysis involving all cerebral compartments (proliferative VZ and SVZ; migratory IZ, and cortical anlage composed of MZ, CP, pSP zone/later expanded SP) using DPN markers, proliferative markers, and fibrillar markers, can serve as a novel important criterion for regional frontal cortex differentiation study. We named it “compartmental histogenesis”.

Using the compartmental approach, the distribution of DPN markers before and during SP expansion phases indirectly suggests that layer VI and the SP share a common developmental origin [48]. During the SP formation phase, the deep CP TBR1+ neurons became spread down, forming a large layer with gradual TBR1+ nuclei spread. At this time, TBR1+/SOX5+ neurons (SOX5 is known as a cortical layer V marker) [21,81,82] maintain their place in the superficial CP portions. In contrast, deep CP parts and gradually expanded SP show the prevalence of TBR1+/CTIP2+ and TBR1+/CELF1+ neurons. By 15 PCW, the expanded SP zone contains numerous TBR1+ cells and shows predominant CELF1 coexpression. The deep portion of secondary condensed CP [5,26,80] corresponds to layer VI coexpresses TBR1+/CTIP2+ markers. We thus propose that layer VI neurons (TBR1+, CTIP2+, and CELF1+) and SP neurons share a common origin at 13 PCW while intermixed in the deep portion of the so-called second CP [80]. One or two weeks later, the expanded SP neuronal population is predominantly TBR1+/CELF1+. After 15 PCW, the difference between layer VI and SP is as follows: SPN has different roles than layer VI neurons at the period of the enormous expansion of SP [5,78], which serves as a waiting compartment for increasing contingent of afferent fibers, especially associative fibers, that become constituents of a large population of interstitial neurons in the gyral white matter of the human cortex.

Importatly, the SP formation is not uniform across the frontal cortical regions. In the orbital cortex, at 13 PCW, there is no gradual loosing of the deep CP, but instead, there is a dense accumulation of TBR1+ nuclei below CP, which give a unique “double plate” appearance. This deep-cell dense band (“double plate”) is continuous with a looser portion of the deep CP in laterodorsal cortical regions. We further showed that “double CP” represents a mixture of late migratory TBR1+ neurons and a downward spread of TBR1+ SP neurons, as the SP became the thickest frontal cortex layer [48,83]. Simultaneous analysis of the entire cerebral wall components with the diverse molecular markers (so-called “compartmental approach”) showed an unexpected SVZ prominence containing the co-expressed TBR1+, TBR2+, and Ki67+ cells. The early SVZ role in the production of projection neurons was not previously elaborated. However, numerous studies emphasized the evolutionary role of late oSVZ in the primate brain [30,84,85] needed for the superficial associative projection neuron production. In previous studies [30,86,87,88,89], authors described significant events in corticogenesis but did not discuss the significance of early SVZ appearance in humans and its role in neuronal production. Rakic [5] and Kostovic [26,90] described that SVZ exists in the early phases of human cortical development. We thus suggest that SVZ prominence during the early cortical development phases, as observed in this study, gives similar significance to the evolutionarily unique characteristics of the human cortex. The reason is as follows: DPN, presumably produced in SVZ, exhibits a number of projections with subcortical structures that are characteristic of primates. In addition, DPN participates in complex cognitive and behavioral functions, such as frontal cortex connectivity with the mediodorsal nucleus and caudate-putamen [39,43]. The projections to the lateral nucleus of the amygdala seem to be human brain characteristics [38]. Considering that SP projection neurons also send their axons transiently in the contralateral and ipsilateral association cortex [91], one can imagine various primate-specific DPN projections.

Therefore, we suggest that the early SVZ performs a similar function as the late SVZ [30,86] by giving rise to a larger number of projection neurons that will be involved in human-characteristic connectivity. Moreover, TBR2 expression in SVZ shows that this early-appearing evolutionarily-new proliferative zone undergoes proliferation activity through the intermediate progenitor cells. However, in the late fetal period, oSVZ, together with fibrilar IZ and expanded SP, may have additional functions, serving as an intermigratory zone for later-formed superficial layer neurons [18,92,93,94]. During the later development, human-characteristic compartments, the SP, and the SVZ may undergo further specifications, such as increased involvement of the SP in the afferent associative fibers compartmentalization (waiting compartment) [5]. The SP may also make synaptic contact with migratory neurons and play a significant role in migratory events [18,94]. Finally, the late SVZ may have an increasing role in glial production [10].

An interesting role of the expanded SP neurons in the projection neuron specialization and subsequent migration into CP was proposed by [95]. Although these authors claim that their finding does not contradict the observation of [96] and [48], our present findings support [48] the concept of SP neurons dispersion but not upwards migration. However, this does not exclude the important role of SP neurons in the interaction and cell-to-cell signaling with migratory neurons [93].

In the present study, we have confirmed previous observations [14,67,68,97] that TBR1, an excellent marker for glutamatergic pyramidal neurons, is also expressed in glutamatergic CRN. Notably, most neurons expressing DPN markers are glutamatergic [98,99]. Besides DPN and SPN, we found that CRN were TBR1+/REELIN+ in all phases of early corticogenesis, suggesting their projection neuron nature and glutamatergic-transmitter profile [97]. CRN plays multiple roles in early connectivity establishment, neural architecture, and REELIN secretion, which is essential for migration regulation [97]. Whether the role of CRN and early phases pioneering SPN [100,101] differ from their prospective morphogenetic role after SP formation (at 13 PCW) is a matter of further research. The sublaminar position of superficially situated CRN and more deeply situated TBR1+ pioneering SPN during the earliest examined phases (late preplate-pioneering CP) supports the idea that they have a role in the early cortical architecture formation. This position favors preplate splitting by incoming new TBR1+ radially migrating neurons.

Previous studies addressed the significant differences and similarities in the cortical development between rodents, primates, and humans [86,87,102,103,104,105,106,107]. Although rats were considered mammals with poorly developed prefrontal cortexes, some authors give evidence of cytoarchitectonics characteristic for the prefrontal cortex even in rodents [108]. However, there is no doubt that the prefrontal cortex is most prominent in primates, especially in humans [46]. In this respect, our findings of early regional differences in the distribution of the TBR1-reactive neurons are difficult to compare directly with finding in rodents. Major similarities in the cortical development between rodents and primates are observed during the earliest phases of corticogenesis, and differences seem to emerge during the later phases of development. These differences in lamination pattern begin to arise after 13–15 PCW during the expansion of the SP and become most prominent during the formation of the primary convolutions when SP achieves its maximum size. At that time, the ratio of the size between SP and CP is 4:1 [5,74,101]. It should be noted that the SP is well developed when all initial transient six compartments are present [28,72], explaining primate-characteristic laminar patterning. Additionally, during this period, the MZ of the human and primate cortex also shows some characteristic features [97]. Regarding specific differences in TBR1 activity in the CP ([68], present study) in humans, it seems that rodent TBR1 activity shows less restricted compartment-specific expression. Human cortical development is characterized by the presence of particularly expanded oSVZ, as reported by [68,87,90]. Moreover, there are cytoarchitectonic distinctions in the proliferative zones in humans and primates represented by the compartmentalization of SVZ [86,87]. Finally, early developmental expression trajectories in humans are more similar to primates than rodents [98] but differ in regional patterning [32,82].

Here, we observed the laminar differences in the early development of SP and CP, which was highly prominent and densely packed during the SP expansion phase. Using the compartmental histogenesis approach and the analysis of the DPN expression patterns through all compartments of the developing neocortex, we discovered additional and specific differences during the early developmental phases of human corticogenesis. Overall, our data suggest that the frontal cortex, besides being well-developed in primates, also displays the early differential distribution of DPN in the human neocortex.

### 4.3. Basic Neural Network Components of Highly Ordered Association Frontal Cortex Differentiate during Early Fetal Period

The laminar DPN markers distribution during the first condensation of CP [5,80] is in agreement with available data on the laminar organization of connectivity elements (axons, dendrites, synapses). Accordingly, the dense DPN packing was present in early condensed CP, while scattered DPN markers+ nuclei were found above (MZ) and below (presubplate, pSP) the CP [5,9,22,42,74]. Central neuronal elements in the early condensed CP represent immature pyramidal neuronal cell bodies, forming the middle layer with connectivity elements distributed above and below the CP [5,9,22,42,74]. In particular, the early-fetal connectivity network within organized laminas is characterized by bilaminar glutamatergic input from the thalamus [42,57,84], cholinergic input from the basal forebrain [109], and monoaminergic modulatory brainstem afferents [110,111]. First, transient synapses in the human cortex were described in the transient MZ and pSP compartments [5,42]. Thus, the early subdivisions (8–13 PCW) in the frontal cortex can be discerned by the differences in the DPN expression. These subdivisions are not only involved in cortical regionalization but also have a functional and prospective morphogenetic role in the establishment of the early frontal-cortex-connectivity network. The first postmigratory neurons, which differentiate into immature pyramidal neurons, show predominantly bipolar dendritic arborization [19,20,69]. Their apical dendrites repeatedly divide within the MZ, forming an immature apical bouquet. On the basal portions of immature pyramidal neurons, the first basal dendrites stretch from the CP to the thin pSP zone [19,20,69]. These pyramidal neuronal cell bodies are densely packed in the first condensed CP. According to the hypothesis by [5] and [48], SP neurons and layer VI neurons are situated within the CP during this early developmental phase. The basic cortical architecture is thus designed by layer VI neurons and SPN with TBR+ nuclei, displaying their postsynaptic dendrites branches within the two synaptic strata [19,42]. This suggests that all inputs from thalamic afferents approach the MZ and the pSP compartments by entering above and below the CP. Such an event provides the first contact between thalamic presynaptic and bilaminar-distributed postsynaptic elements in the early fetal cortex. This relationship between radially-oriented cell geometry and incoming early afferents is favorable for other cortical afferents arriving at the frontal cortex. The bilaminar afferent input is important for the earliest contacts with superficially positioned CRN and pioneering SPN that originate from the deeper preplate sublayer and later represent postsynaptic elements in the pSP [5,101]. On the other hand, DPN axons from the early CP advance quickly towards the subcortical centers since they reach the pons and spinal cord by 15 PCW [47]. The early intrinsic circuitry with local circuitry neurons participation is less examined because it is challenging to trace axonal arborization of early GABAergic neurons containing calretinin, parvalbumin, somatostatin, VIP, NPY, and other peptidergic markers. However, the presence of calretinin (CALR)+ [97,112] early during development, as well as during the SP formation period [10], suggests that these interneuronal populations may form cell-to-cell contacts in a synaptic or non-synaptic fashion. Simultaneously, CP predominantly consists of DPN since the superficial projection neurons are still not being produced [23,64]. With these extrinsic (afferent and efferent) [113] and intrinsic (interneuronal circuitry) connections [114,115,116], TBR1+ DPN establishes the basic framework for cortical connectivity. The early pyramidal neurons and differentiation of the neuronal circuitries in the association prefrontal cortex clearly indicate that neuronal networks, necessary for complex cognitive, executive, and behavioral functions, begin to develop early in fetal life. This is in agreement with the experimental data on synaptogenesis in the primate frontal cortex [117] and early SP differentiation as the main synaptic compartment [5,70,118,119]. Additionally, such observations support the recent findings on in vivo in utero functional connectivity [120,121,122], showing that frontal networks develop in parallel with the other emerging functional resting-state connectivity. Moreover, frontal networks show early lateralization [121]. Recent studies also suggest that the fetal SP-centered functional state is one of the two active principal networks in the human fetal brain. Thus, the most prominent SP of the prefrontal cortex may have a significant impact on the global fetal cortical connectome development. The SP network is the first network to respond to sensory stimuli [123], and its tangential nexus provides continuity between the transient fetal and the permanent adult networks [101]. During perinatal and early postnatal life, synaptogenesis in the prefrontal cortex occurs with similar intensity as in primary sensory and motor-cortical areas [124,125]. However, the refinement of the cortical microstructure occurs earlier and more rapidly in the sensorimotor cortices than in the association cortices [126].

In conclusion, all connectivity data are consistent with the concept that early fetal life is a critical period for the development of a highly-ordered association cortex [31,34,38]. Intensive differentiation periods are vulnerable to different genetic and epigenetic factors [30,31,34,107,127,128]. Evidently, lesions during the critical period may have profound consequences on further prefrontal circuitry development and can eventually lead to the development of neuro-cognitive disorders, such as schizophrenia and autism [129,130], and behavioral disorders, such as ADHD [131].

## Figures and Tables

**Table 1:** List of antibodies used in the study.

**Primary antibodies**				
Antibody	type	Cat. N^o^	Supplier	Dilution
**CELF1** (CUGBP1)	mouse monoclonal	sc-20003	Santa Cruz biotechnology	1:250
**CELF1** (CUGBP1)	rabbit polyclonal	ab129115	Abcam	1:1000
**TBR1**	rabbit polyclonal	ab31940	Abcam	1:150
**TBR2**	rabbit polyclonal	ab23345	Abcam (RT)	1:200
**CTIP2**	rat monoclonal	ab18465	Abcam	1:500
**TLE4**	mouse monoclonal	sc-365406	Santa Cruz biotechnology	1:50
**SOX5**	rabbit polyclonal	ab94396	Abcam	1:1000
**RELN**	mouse monoclonal	MAB5366	Millipore	1:1000
**CALR**	mouse monoclonal	6B3	Swant	1:1000
**GAD67**	mouse monoclonal	MAB5406	Millipore	1:500
**DCX**	mouse monoclonal	sc-271390	Santa Cruz biotechnology	1:50
**PAX6**	mouse monoclonal	AMAb91372	Atlas Antibodies	1:500
**TUBB3**	rabbit polyclonal	PRB-435P	BioLegend	1:1000
**SMI312**	mouse monoclonal	837901	BioLegend	1:1000
**Ki67**	mouse monoclonal	M7240	Dako	1:50
**Secondary antibodies**				
**Alexa Fluor 488**	goat anti-rabbit	A-32731	Thermo Fisher Scientific	1:1000
**Alexa Fluor 555**	goat anti-mouse	A-21422	Thermo Fisher Scientific	1:1000
**Alexa Fluor 555**	goat anti-rat	A-21434	Thermo Fisher Scientific	1:1000

## Data Availability

All the data reported are available on request from the corresponding author.

## References

[1] His W. (1904). Die Entwicklung des Menschlichen Gehirns Wahrend der Ersten Monate.

[2] Von Economo C.B., Koskinas G.N. (1925). Atlas of Cytoarchitectonics of the Adult Human Cerebral Cortex.

[3] Rose M. (1926). Uber das histogenetische Prinzip der Einteilung der Grosshirnrinde. J. Psychol. Neurol..

[4] Filimonoff I.N. (1947). A Rational Subdivision of the Cerebral Cortex. Arch. Neurol. Psych..

[5] Kostovic I., Rakic P. (1990). Developmental History of the Transient Subplate Zone in the Visual and Somatosensory Cortex of the Macaque Monkey and Human Brain. J. Comp. Neurol..

[6] Meyer G., Schaaps J.P., Moreau L., Goffinet A.M. (2000). Embryonic and Early Fetal Development of the Human Neocortex. J. Neurosci..

[7] Meyer G. (2001). Human neocortical development: The importance of embryonic and early fetal events. Neuroscientist..

[8] Bystron I., Blakemore C., Rakic P. (2008). Development of the Human Cerebral Cortex: Boulder Committee Revisited. Nat. Rev. Neurosci..

[9] Kostović I., Judaš M., Toga A.W. (2015). Embryonic and Fetal Development of the Human Cerebral Cortex. Brain Mapping: An Encyclopedic Reference.

[10] Kostović I., Išasegi I.Ž., Krsnik Ž. (2019). Sublaminar Organization of the Human Subplate: Developmental Changes in the Distribution of Neurons, Glia, Growing Axons and Extracellular Matrix. J. Anat..

[11] Clascá F., Angelucci A., Sur M. (1995). Layer-Specific Programs of Development in Neocortical Projection Neurons. Proc. Natl. Acad. Sci. USA.

[12] Han W., Kwan K.Y., Shim S., Lam M.M.S., Shin Y., Xu M., Zhu Y., Li M., Šestan N. (2011). TBR1 Directly Represses Fezf2 to Control the Laminar Origin and Development of the Corticospinal Tract. Proc. Natl. Acad. Sci. USA.

[13] Kwan K.Y., Šestan N., Anton E.S. (2012). Transcriptional Co-Regulation of Neuronal Migration and Laminar Identity in the Neocortex. Development.

[14] Hevner R.F., Shi L., Justice N., Hsueh Y.P., Sheng M., Smiga S., Bulfone A., Goffinet A.M., Campagnoni A.T., Rubenstein J.L.R. (2001). Tbr1 Regulates Differentiation of the Preplate and Layer 6. Neuron.

[15] Arlotta P., Molyneaux B.J., Chen J., Inoue J., Kominami R., MacKlis J.D. (2005). Neuronal Subtype-Specific Genes That Control Corticospinal Motor Neuron Development In Vivo. Neuron.

[16] Molyneaux B.J., Arlotta P., Menezes J.R.L., Macklis J.D. (2007). Neuronal Subtype Specification in the Cerebral Cortex. Nat. Rev. Neurosci..

[17] Greig L.C., Woodworth M.B., Galazo M.J., Padmanabhan H., Macklis J.D. (2013). Molecular Logic of Neocortical Projection Neuron Specification, Development and Diversity. Nat. Rev. Neurosci..

[18] Ohtaka-Maruyama C., Okamoto M., Endo K., Oshima M., Kaneko N., Yura K., Okado H., Miyata T., Maeda N. (2018). Synaptic Transmission from Subplate Neurons Controls Radial Migration of Neocortical Neurons. Science.

[19] Marin-Padilla M. (1983). Structural Organization of the Human Cerebral Cortex Prior to the Appearance of the Cortical Plate. Anat. Embryol..

[20] Mrzljak L., Uylings H.B.M., Kostović I., Van Eden C.G. (1988). Prenatal development of neurons in the human prefrontal cortex. I: A qualitative Golgi study. J. Comp. Neurol..

[21] Kwan K.Y., Lam M.M.S., Krsnik Ž., Kawasawa Y.I., Lefebvre V., Šestan N. (2008). SOX5 Postmitotically Regulates Migration, Postmigratory Differentiation, and Projections of Subplate and Deep-Layer Neocortical Neurons. Proc. Natl. Acad. Sci. USA.

[22] Dubois J., Kostovic I., Judas M., Toga A.W. (2015). Development of Structural and Functional Connectivity. Brain Mapping: An Encyclopedic Reference.

[23] Rakic P. (1988). Specification of cerebral cortical areas. Science.

[24] Popovitchenko T., Park Y., Page N.F., Luo X., Krsnik Z., Liu Y., Salamon I., Stephenson J.D., Kraushar M.L., Volk N.L. (2020). Translational Derepression of Elavl4 Isoforms at Their Alternative 5′ UTRs Determines Neuronal Development. Nat. Commun..

[25] Salamon I., Rasin M.R. (2022). Evolution of the Neocortex Through RNA-Binding Proteins and Post-Transcriptional Regulation. Front. Neurosci..

[26] Kostovic I., Hinrichsen K.V., Beier H.M., Breucker H., Christ B., Duncker H.R., Dvořák M., Gaudecker B., Dorsche H.H., Holstein A.F. (1990). Zentralnervensystem. Humanembryologie: Lehrbuch Und Atlas Der Vorgwburtlichen Entwicklung Des Menschen.

[27] Kast R.J., Levitt P. (2019). Precision in the Development of Neocortical Architecture: From Progenitors to Cortical Networks. Prog. Neurobiol..

[28] Kostović I., Judaš M. (2002). Correlation between the sequential ingrowth of afferents and transient patterns of cortical lamination in preterm infants. Anat Rec..

[29] Silbereis J.C., Pochareddy S., Zhu Y., Li M., Sestan N. (2016). The Cellular and Molecular Landscapes of the Developing Human Central Nervous System. Neuron.

[30] Nowakowski T.J., Bhaduri A., Pollen A.A., Alvarado B., Mostajo-Radji M.A., Di Lullo E., Haeussler M., Sandoval-Espinosa C., Liu S.J., Velmeshev D. (2017). Spatiotemporal Gene Expression Trajectories Reveal Developmental Hierarchies of the Human Cortex. Science.

[31] Sestan N., State M.W. (2018). Lost in Translation: Traversing the Complex Path from Genomics to Therapeutics in Autism Spectrum Disorder. Neuron.

[32] Cadwell C.R., Bhaduri A., Mostajo-Radji M.A., Keefe M.G., Nowakowski T.J. (2019). Development and Arealization of the Cerebral Cortex. Neuron.

[33] Miller J.A., Ding S.L., Sunkin S.M., Smith K.A., Ng L., Szafer A., Ebbert A., Riley Z.L., Royall J.J., Aiona K. (2014). Transcriptional Landscape of the Prenatal Human Brain. Nature.

[34] Polioudakis D., de la Torre-Ubieta L., Langerman J., Elkins A.G., Shi X., Stein J.L., Vuong C.K., Nichterwitz S., Gevorgian M., Opland C.K. (2019). A Single Cell Transcriptomic Atlas of Human Neocortical developmentduring Mid-Gestation. Neuron.

[35] Willsey A.J., Sanders S.J., Li M., Dong S., Tebbenkamp A.T., Muhle R.A., Reilly S.K., Lin L., Fertuzinhos S., Miller J.A. (2013). Coexpression Networks Implicate Human Midfetal Deep Cortical Projection Neurons in the Pathogenesis of Autism. Cell.

[36] Fazel Darbandi S., Robinson Schwartz S.E., Qi Q., Catta-Preta R., Pai E.L.L., Mandell J.D., Everitt A., Rubin A., Krasnoff R.A., Katzman S. (2018). Neonatal Tbr1 Dosage Controls Cortical Layer 6 Connectivity. Neuron.

[37] Molnár Z., Luhmann H.J., Kanold P.O. (2020). Transient Cortical Circuits Match Spontaneous and Sensory-Driven Activity during Development. Science.

[38] Nauta W.J. (1972). Neural associations of the frontal cortex. Acta Neurobiol. Exp..

[39] Goldman P.S., Nauta W.J.H. (1977). An Intricately Patterned Prefronto-Caudate Projection in the Rhesus Monkey. J. Comp. Neurol..

[40] Stephan H., Stephan H. (1975). Mikroskopische Anatomie. Allocortex.

[41] Kahle W. (1969). Die Entwicklung Der Menschlichen Großhirnhemisphäre.

[42] Molliver M.E., Kostović I., Van Der Loos H. (1973). The Development of Synapses in Cerebral Cortex of the Human Fetus. Brain Res..

[43] Kostovic I., Goldman-Rakic P.S. (1983). Transient Cholinesterase Staining in the Mediodorsal Nucleus of the Thalamus and Its Connections in the Developing Human and Monkey Brain. J. Comp. Neurol..

[44] Goldman-Rakic P.S. (1987). Development of cortical circuitry and cognitive function. Child Dev..

[45] Fuster J.M. (2008). The Prefrontal Cortex.

[46] Kolk S.M., Rakic P. (2021). Development of Prefrontal Cortex. Neuropsychopharmacology.

[47] Eyre J.A., Clowry G.J. (2002). Development of the human spinal cord. Brain.

[48] Duque A., Krsnik Z., Kostović I., Rakic P. (2016). Secondary Expansion of the Transient Subplate Zone in the Developing Cerebrum of Human and Nonhuman Primates. Proc. Natl. Acad. Sci. USA.

[49] Englund C., Fink A., Lau C., Pham D., Daza R.A.M., Bulfone A., Kowalczyk T., Hevner R.F. (2005). Pax6, Tbr2, and Tbr1 Are Expressed Sequentially by Radial Glia, Intermediate Progenitor Cells, and Postmitotic Neurons in Developing Neocortex. J. Neurosci..

[50] Ip B.K., Bayatti N., Howard N.J., Lindsay S., Clowry G.J. (2011). The Corticofugal Neuron-Associated Genes ROBO1, SRGAP1, and CTIP2 Exhibit an Anterior to Posterior Gradient of Expression in Early Fetal Human Neocortex Development. Cereb. Cortex.

[51] Bulfone A., Smiga S.M., Shimamura K., Peterson A., Puelles L., Rubenstein J.L.R. (1995). T-Brain-1: A Homolog of Brachyury Whose Expression Defines Molecularly Distinct Domains within the Cerebral Cortex. Neuron.

[52] Kolk S.M., Whitman M.C., Yun M.E., Shete P., Donoghue M.J. (2006). A Unique Subpopulation of Tbr1-Expressing Deep Layer Neurons in the Developing Cerebral Cortex. Mol. Cell. Neurosci..

[53] Aboitiz F., Montiel J., García R.R. (2005). Ancestry of the Mammalian Preplate and Its Derivatives: Evolutionary Relicts or Embryonic Adaptations?. Rev. Neurosci..

[54] O’Rahilly R., Müller F. (2008). Significant Features in the Early Prenatal Development of the Human. Brain. Ann. Anat..

[55] Papadopulos F., Spinelli M., Valente S., Foroni L., Orrico C., Alviano F., Pasquinelli G. (2007). Common Tasks in Microscopic and Ultrastructural Image Analysis Using ImageJ. Ultrastruct. Pathol..

[56] Grishagin I.V. (2015). Automatic Cell Counting with ImageJ. Anal. Biochem..

[57] Marin-Padilla M. (1970). Prenatal and Early Postnatal Ontogenesis of the Human Motor Cortex: A Golgi Study. I. The Sequential Development of the Cortical Layers. Brain Res..

[58] O’Leary D.D.M., Chou S.J., Sahara S. (2007). Area Patterning of the Mammalian Cortex. Neuron.

[59] Chou S.J., Babot Z., Leingärtner A., Studer M., Nakagawa Y., O’Leary D.D.M. (2013). Geniculocortical Input Drives Genetic Distinctions between Primary and Higher-Order Visual Areas. Science.

[60] Simi A., Studer M. (2018). Developmental Genetic Programs and Activity-Dependent Mechanisms Instruct Neocortical Area Mapping. Curr. Opin. Neurobiol..

[61] O’Leary D.D.M. (1989). Do Cortical Areas Emerge from a Protocortex?. Trends Neurosci..

[62] Rakic P. (2000). Radial Unit Hypothesis of Neocortical Expansion. Novartis Found. Symp..

[63] Fukuchi-Shimogori T., Grove E.A. (2001). Neocortex Patterning by the Secreted Signaling Molecute FGF8. Science.

[64] Rakic P., Ayoub A.E., Breunig J.J., Dominguez M.H. (2009). Decision by Division: Making Cortical Maps. Trends Neurosci..

[65] Kraushar M.L., Viljetic B., Wijeratne H.R.S., Thompson K., Jiao X., Pike J.W., Medvedeva V., Groszer M., Kiledjian M., Hart R.P. (2015). Thalamic WNT3 Secretion Spatiotemporally Regulates the Neocortical Ribosome Signature and MRNA Translation to Specify Neocortical Cell Subtypes. J. Neurosci..

[66] Luhmann H.J., Khazipov R. (2018). Neuronal Activity Patterns in the Developing Barrel Cortex. Neuroscience.

[67] Bystron I., Rakic P., Molnár Z., Blakemore C. (2006). The First Neurons of the Human Cerebral Cortex. Nat. Neurosci..

[68] Bayatti N., Sarma S., Shaw C., Eyre J.A., Vouyiouklis D.A., Lindsay S., Clowry G.J. (2008). Progressive Loss of PAX6, TBR2, NEUROD and TBR1 MRNA Gradients Correlates with Translocation of EMX2 to the Cortical Plate during Human Cortical Development. Eur. J. Neurosci..

[69] Kostović-Knežević L., Kostović I., Krmpotić-Nemanić J., Kelović Z., Vuković B. (1978). The cortical plate of the human neocortex during the early fetal period (at 31–65 mm CRL). Verh. Anat. Ges..

[70] Molnár Z., Rockland K.S., Rubenstein J., Rakic P., Chen B., Kwan K.Y. (2020). Cortical columns. Neural Circuit and Cognitive Development.

[71] Marín-Padilla M. (1992). Ontogenesis of the pyramidal cell of the mammalian neocortex and developmental cytoarchitectonics: A unifying theory. J. Comp. Neurol..

[72] Brodmann K. (1909). Vergleichende Lokalisationslehre der Grosshirnrinde in Ihren Prinzipien Dargestellt auf Grund des Zellenbaues.

[73] Bayer S.A., Altman J. (2007). The Human Brain During the Early First Trimester.

[74] Kostović I., Sedmak G., Judaš M. (2019). Neural Histology and Neurogenesis of the Human Fetal and Infant Brain. Neuroimage.

[75] Žunić Išasegi I., Radoš M., Krsnik Ž., Radoš M., Benjak V., Kostović I. (2018). Interactive Histogenesis of Axonal Strata and Proliferative Zones in the Human Fetal Cerebral Wall. Brain Struct. Funct..

[76] Haydar T.F., Wang F., Schwartz M.L., Rakic P. (2000). Differential Modulation of Proliferation in the Neocortical Ventricular and Subventricular Zones. J. Neurosci..

[77] Haubensak W., Attardo A., Denk W., Huttner W.B. (2004). Neurons Arise in the Basal Neuroepithelium of the Early Mammalian Telencephalon: A Major Site of Neurogenesis. Proc. Natl. Acad. Sci. USA.

[78] Vasung L., Lepage C., Radoš M., Pletikos M., Goldman J.S., Richiardi J., Raguž M., Fischi-Gómez E., Karama S., Huppi P.S. (2016). Quantitative and Qualitative Analysis of Transient Fetal Compartments during Prenatal Human Brain Development. Front. Neuroanat..

[79] Osheroff H., Hatten M.E. (2009). Gene Expression Profiling of Preplate Neurons Destined for the Subplate: Genes Involved in Transcription, Axon Extension, Neurotransmitter Regulation, Steroid Hormone Signaling, and Neuronal Survival. Cereb. Cortex.

[80] Poliakov G.I., Sarkisov A.S., Filimonof I.N., Preobrazenskaya N.S. (1949). Structural organization of the human cerebral cortex during ontogenetic development. Cytoarchitectonics of the Cerebral Cortex in Man.

[81] Hevner R.F. (2007). Layer-Specific Markers as Probes for Neuron Type Identity in Human Neocortex and Malformations of Cortical Development. J. Neuropathol. Exp. Neurol..

[82] Bedogni F., Hodge R.D., Elsen G.E., Nelson B.R., Daza R.A.M., Beyer R.P., Bammler T.K., Rubenstein J.L.R., Hevner R.F. (2010). Tbr1 Regulates Regional and Laminar Identity of Postmitotic Neurons in Developing Neocortex. Proc. Natl. Acad. Sci. USA.

[83] Kostović I. (1991). Structural and histochemical reorganization of the human prefrontal cortex during perinatal and postnatal life. Prog. Brain Res..

[84] Kelava I., Reillo I., Murayama A.Y., Kalinka A.T., Stenzel D., Tomancak P., Matsuzaki F., Lebrand S.E., Schwamborn J.C., Okano H. (2012). Abundant occurrence of basal radial glia in the subventricular zone of embryonic neocortex of a lissencephalic primate, the common marmoset callithrix jacchus. Cereb. Cortex.

[85] Kalebic N., Huttner W.B. (2020). Basal Progenitor Morphology and Neocortex Evolution. Trends Neurosci..

[86] Smart I.H.M., Dehay C., Giroud P., Berland M., Kennedy H. (2002). Unique Morphological Features of the Proliferative Zones and Postmitotic Compartments of the Neural Epithelium Giving Rise to Striate and Extrastriate Cortex in the Monkey. Cereb. Cortex.

[87] Molnár Z., Clowry G. (2012). Cerebral Cortical Development in Rodents and Primates. Prog. Brain Res..

[88] Kriegstein A., Noctor S., Martínez-Cerdeño V. (2006). Patterns of neural stem and progenitor cell division may underlie evolutionary cortical expansion. Nat. Rev. Neurosci..

[89] Rakic P. (2009). Evolution of the neocortex: A perspective from developmental biology. Nat. Rev. Neurosci..

[90] Zecevic N., Chen Y., Filipovic R. (2005). Contributions of cortical subventricular zone to the development of the human cerebral cortex. J. Comp. Neurol..

[91] DeAzevedo L.C., Hedin-Pereira C., Lent R. (1997). Callosal neurons in the cingulate cortical plate and subplate of human fetuses. J. Comp. Neurol..

[92] Sidman R.L., Rakic P. (1973). Neuronal Migration, with Special Reference to Developing Human Brain: A Review. Brain Res..

[93] Kostović I., Sedmak G., Vukšić M., Judaš M. (2015). The Relevance of Human Fetal Subplate Zone for Developmental Neuropathology of Neuronal Migration Disorders and Cortical Dysplasia. CNS Neurosci. Ther..

[94] Ohtaka-Maruyama C. (2020). Subplate Neurons as an Organizer of Mammalian Neocortical Development. Front. Neuroanat..

[95] Ozair M.Z., Kirst C., van den Berg B.L., Ruzo A., Rito T., Brivanlou A.H. (2018). HPSC Modeling Reveals That Fate Selection of Cortical Deep Projection Neurons Occurs in the Subplate. Cell Stem Cell.

[96] Rakic P. (1974). Neurons in Rhesus Monkey Visual Cortex: Systematic Relation between Time of Origin and Eventual Disposition. Science.

[97] Meyer G., González-Gómez M. (2018). The Subpial Granular Layer and Transient Versus Persisting Cajal-Retzius Neurons of the Fetal Human Cortex. Cereb. Cortex.

[98] Bakken T.E., Miller J.A., Ding S.L., Sunkin S.M., Smith K.A., Ng L., Szafer A., Dalley R.A., Royall J.J., Lemon T. (2016). A Comprehensive Transcriptional Map of Primate Brain Development. Nature.

[99] Berg J., Sorensen S.A., Ting J.T., Miller J.A., Chartrand T., Buchin A., Bakken T.E., Budzillo A., Dee N., Ding S.L. (2021). Human Neocortical Expansion Involves Glutamatergic Neuron Diversification. Nature.

[100] McConnell S.K., Ghosh A., Shatz C.J. (1989). Subplate Neurons Pioneer the First Axon Pathway from the Cerebral Cortex. Science.

[101] Kostović I. (2020). The Enigmatic Fetal Subplate Compartment Forms an Early Tangential Cortical Nexus and Provides the Framework for Construction of Cortical Connectivity. Prog. Neurobiol..

[102] Lickiss T., Cheung A.F.P., Hutchinson C.E., Taylor J.S.H., Molnár Z. (2012). Examining the relationship between early axon growth and transcription factor expression in the developing cerebral cortex. J. Anat..

[103] Miller D.J., Bhaduri A., Sestan N., Kriegstein A. (2019). Shared and derived features of cellular diversity in the human cerebral cortex. Curr. Opin. Neurobiol..

[104] Bayer S.A., Altman J. (1990). Development of Layer I and the Subplate in the Rat Neocortex. Exp. Neurol..

[105] Molnár Z., Métin C., Stoykova A., Tarabykin V., Price D.J., Francis F., Meyer G., Dehay C., Kennedy H. (2006). Comparative aspects of cerebral cortical development. Eur. J. Neurosci..

[106] Cheung A.F., Pollen A.A., Tavare A., DeProto J., Molnár Z. (2007). Comparative aspects of cortical neurogenesis in vertebrates. J. Anat..

[107] Ma S., Skarica M., Li Q., Xu C., Risgaard R.D., Tebbenkamp A.T.N., Mato-Blanco X., Kovner R., Krsnik Ž., de Martin X. (2022). Molecular and cellular evolution of the primate dorsolateral prefrontal cortex. Science.

[108] Van Eden C.G., Uylings H.B.M. (1985). Cytoarchitectonic Development of the Prefrontal Cortex in the Rat. J. Comp. Neurol..

[109] Kostović I. (1986). Prenatal Development of Nucleus Basalis Complex and Related Fiber Systems in Man: A Histochemical Study. Neuroscience.

[110] Nobin A., Björklund A. (1973). Topography of the Monoamine Neuron Systems in the Human Brain as Revealed in Fetuses. Acta Physiol. Scand..

[111] Zecevic N., Verney C. (1995). Development of the Catecholamine Neurons in Human Embryos and Fetuses, with Special Emphasis on the Innervation of the Cerebral Cortex. J. Comp. Neurol..

[112] Meyer G., González-Arnay E., Moll U., Nemajerova A., Tissir F., González-Gómez M. (2019). Cajal-Retzius neurons are required for the development of the human hippocampal fissure. J. Anat..

[113] McKenna W.L., Betancourt J., Larkin K.A., Abrams B., Guo C., Rubenstein J.L.R., Chen B. (2011). Tbr1 and Fezf2 Regulate Alternate Corticofugal Neuronal Identities during Neocortical Development. J. Neurosci..

[114] Ben-Ari Y., Khalilov I., Represa A., Gozlan H. (2004). Interneurons Set the Tune of Developing Networks. Trends Neurosci..

[115] Jakovcevski I., Mayer N., Zecevic N. (2010). Multiple Origins of Human Neocortical Interneurons Are Supported by Distinct Expression of Transcription Factors. Cereb. Cortex.

[116] Ma T., Wang C., Wang L., Zhou X., Tian M., Zhang Q., Zhang Y., Li J., Liu Z., Cai Y. (2013). Subcortical Origins of Human and Monkey Neocortical Interneurons. Nat. Neurosci..

[117] Bourgeois J.P., Goldman-Rakic P.S., Rakic P. (1994). Synaptogenesis in the Prefrontal Cortex of Rhesus Monkeys. Cereb. Cortex.

[118] Kostović I., Radoš M., Kostović-Srzentić M., Krsnik Ž. (2021). Fundamentals of the Development of Connectivity in the Human Fetal Brain in Late Gestation: From 24 Weeks Gestational Age to Term. J. Neuropathol. Exp. Neurol..

[119] Hoerder-Suabedissen A., Molnár Z. (2015). Development, Evolution and Pathology of Neocortical Subplate Neurons. Nat. Rev. Neurosci..

[120] Thomason M.E., Grove L.E., Lozon T.A., Vila A.M., Ye Y., Nye M.J., Manning J.H., Pappas A., Hernandez-Andrade E., Yeo L. (2015). Age-Related Increases in Long-Range Connectivity in Fetal Functional Neural Connectivity Networks in Utero. Dev. Cogn. Neurosci..

[121] Kim J.H., De Asis-Cruz J., Cook K.M., Limperopoulos C. (2022). Gestational Age-Related Changes in the Fetal Functional Connectome: In Utero Evidence for the Global Signal. Cereb. Cortex.

[122] Karolis V., Fitzgibbon S., Cordero-Grande L., Farahibozorg R., Price A., Hughes E., Fetit A., Kariakopoulou V., Pietsch M., Rutherford M. (2022). Maturational Networks of Fetal Brain Activity. bioRxiv.

[123] Wess J.M., Isaiah A., Watkins P.V., Kanold P.O. (2017). Subplate Neurons Are the First Cortical Neurons to Respond to Sensory Stimuli. Proc. Natl. Acad. Sci. USA.

[124] Rakic P., Bourgeois J.P., Eckenhoff M.F., Zecevic N., Goldman-Rakic P.S. (1986). Concurrent Overproduction of Synapses in Diverse Regions of the Primate Cerebral Cortex. Science.

[125] Zecevic N. (1998). Synaptogenesis in layer I of the human cerebral cortex in the first half of gestation. Cereb. Cortex.

[126] Nielsen A.N., Kaplan S., Meyer D., Alexopoulos D., Kenley J.K., Smyser T.A., Wakschlag L.S., Norton E.S., Raghuraman N., Warner B.B. (2022). Maturation of Large-Scale Brain Systems over the First Month of Life. Cereb. Cortex.

[127] Molnár Z., Rutherford M. (2013). Brain Maturation after Preterm Birth. Sci. Transl. Med..

[128] Huttenlocher P.R. (1999). Dendritic and synaptic development in human cerebral cortex: Time course and critical periods. Dev. Neuropsychol..

[129] Weinberger D.R. (1987). Implications of Normal Brain Development for the Pathogenesis of Schizophrenia. Arch. Gen. Psychiatry.

[130] Kostović I., Judaš M., Sedmak G. (2011). Developmental History of the Subplate Zone, Subplate Neurons and Interstitial White Matter Neurons: Relevance for Schizophrenia. Int. J. Dev. Neurosci..

[131] Marchand W.R. (2010). Cortico-Basal Ganglia Circuitry: A Review of Key Research and Implications for Functional Connectivity Studies of Mood and Anxiety Disorders. Brain Struct. Funct..

